# Assessment of the Spatial and Temporal Variations of Water Quality for Agricultural Lands with Crop Rotation in China by Using a HYPE Model

**DOI:** 10.3390/ijerph13030336

**Published:** 2016-03-18

**Authors:** Yunxing Yin, Sanyuan Jiang, Charlotta Pers, Xiaoying Yang, Qun Liu, Jin Yuan, Mingxing Yao, Yi He, Xingzhang Luo, Zheng Zheng

**Affiliations:** 1 College of Environmental Science and Engineering, Fudan University, Shanghai 200433, China; 13210740032@fudan.edu.cn (Y.Y.); xiaoying@fudan.edu.cn (X.Y.); 13210740046@fudan.edu.cn (J.Y.); 13210740033@fudan.edu.cn (M.Y.); zzhenghj@fudan.edu.cn (Z.Z.); 2 Nanjing Institute of Geography and Limnology, Nanjing 210008, China; syjiang@niglas.ac.cn; 3 Swedish Meteorological and Hydrological Institute, Norrkoping SE-601 76, Sweden; Charlotta.Pers@smhi.se; 4 Zhumadian City Environmental Protection Bureau, Zhuamadian 463000, China; zmdliuqun@163.com; 5 Tyndall Centre for Climate Change Research, School of Environmental Sciences, University of East Anglia, Norwich , Norfolk NR4 7TJ, UK; yi.he@uea.ac.uk

**Keywords:** HYPE model, agricultural lands, multi-site and multi-objective calibration, nutrient modeling, crop rotation

## Abstract

Many water quality models have been successfully used worldwide to predict nutrient losses from anthropogenically impacted catchments, but hydrological and nutrient simulations with limited data are difficult considering the transfer of model parameters and complication of model calibration and validation. This study aims: (i) to assess the performance capabilities of a new and relatively more advantageous model, namely, Hydrological Predictions for the Environment (HYPE), that simulates stream flow and nutrient load in agricultural areas by using a multi-site and multi-objective parameter calibration method and (ii) to investigate the temporal and spatial variations of total nitrogen (TN) and total phosphorous (TP) concentrations and loads with crop rotation by using the model for the first time. A parameter estimation tool (PEST) was used to calibrate parameters. Results show that the parameters related to the effective soil porosity were highly sensitive to hydrological modeling. N balance was largely controlled by soil denitrification processes. P balance was influenced by the sedimentation rate and production/decay of P in rivers and lakes. The model reproduced the temporal and spatial variations of discharge and TN/TP relatively well in both calibration (2006–2008) and validation (2009–2010) periods. Among the obtained data, the lowest Nash-Suttclife efficiency of discharge, daily TN load, and daily TP load were 0.74, 0.51, and 0.54, respectively. The seasonal variations of daily TN concentrations in the entire simulation period were insufficient, indicated that crop rotation changed the timing and amount of N output. Monthly TN and TP simulation yields revealed that nutrient outputs were abundant in summer in terms of the corresponding discharge. The area-weighted TN and TP load annual yields in five years showed that nutrient loads were extremely high along Hong and Ru rivers, especially in agricultural lands.

## 1. Introduction

Eutrophication is caused by excessive inputs of nitrogen (N) and phosphorus (P) in rivers and lakes; as such, this phenomenon has been extensively investigated [1,2,3,4,5]. P and N can be readily transported in streamflows and their concentrations are highly dependent on land use in catchments [6]. P and/or N inputs should be reduced to solve eutrophication problems [7]. Point sources, such as sewage plants, and diffuse sources, such as fertilizer and manure, are the main causes of nutrient pollution in water bodies.

The Hong–Ru River basin located in the mid-eastern China is representative of agricultural areas. However, the water quality of Hong–Ru River Basin does not satisfy the expected category III criteria, which are 1.0 mg/L and 0.2 mg/L for total nitrogen (TN) and total phosphorous (TP), respectively, as recommended by the Chinese Environmental Quality Standards for Surface Water [8]. Therefore, long-term discharge and water quality trends should be assessed to help determine potential water quality problems. Probable causes, such as human interventions, should also be identified to provide useful information and references for an enhanced water resource management [9]. The agricultural landscape should be subjected to a case study because of its similarity to other basins with intensively managed agricultural landscapes.

Hydrological processes are strongly dependent on climate change, topography, soil properties, and catchment scale [10,11]. Hydrological transport greatly influences N export, while N concentrations in stream water are mainly controlled by shallow subsurface flow [10,11,12]. N loads are greatly affected by N leaching from different soils, transformations, and storage processes during lateral transport in surface water [13]. Human activities also influence nutrient (e.g., TN and TP) concentrations because of hydrological and ecological interactions. Previous studies on managed catchments revealed a proportional relationship between discharge and nutrient leaching load; this finding confirms that nutrient leaching is transport limited rather than supply limited [14,15,16]. Stream nutrient loads are also greatly affected by agricultural applications and land use [13,17]. Different land use types determine the amount and timing of fertilizer inputs in a covered area. Precipitation and temperature influence nitrate leaching via runoff generation and nutrient turnover processes [18,19,20,21]. In general, basins with sparse data cannot be readily applied to process-based models [22]. Water quality sampling is often insufficient in terms of temporal resolution compared with discharge observations because of financial and personal constraints [10,23]. Thus, interpolation approaches should be considered when we estimate nutrient loads over a relatively long period by using existing gauged points in a specific region [22,24].

A few well-known models, such as SWAT [25], SWRRB [26], and ANSWERS [27], have been developed to simulate hydrological and water quality. However, SWRRB and ANSWERS are based on oversimplified underlying hydrological structures [21]. As the most widely used model, SWAT has several disadvantages; for instance, this model requires a large amount of input data and disregards groundwater entering deep aquifers during hydrological simulations [28,29]. Moreover, SWAT requires intensive parameter calibration [21]. To overcome this problem, the Swedish Meteorological and Hydrological Institute (SMHI) developed Hydrological Predictions for the Environment (HYPE) between 2005 and 2007; HYPE is a process-based, semi-distributed hydrological water quality model established on the basis of the HBV-NP water quality model [30,31,32,33]. The HYPE model simulates stream flow and substances, such as N and P, from precipitation to transportation through soil, rivers, lakes, and river outlets [32]. Thus far, this model has been used successfully in several regions. For example, TN and TP simulations are consistent with observations in two large basins in southern Sweden by transferring calibrated parameters in test basins [32]. The model also demonstrates the temporal and spatial variations of long-term average discharge and nutrient concentrations in Sweden with an area of approximately 450,000 km^2^ [34] and in nested mesoscale catchments in central Germany [10]. However, the model has not been tested in agricultural areas with crop rotation under different climate and physiographic characteristics. Therefore, this study aimed to extend the application of HYPE in agricultural areas.

In this study, a parameter estimation tool (PEST) was used to calibrate hydrological and water quality parameters simultaneously; this multi-objective calibration is more appropriate than other calibration methods to increase constraints on hydrological and water quality processes because both processes are related to each other [11,12,14,21,35]. This study aimed: (i) to assess the performance capabilities of the HYPE model to simulate stream flow and nutrient load in an agricultural area by using a multi-site and multi-objective calibration method and (ii) to investigate the temporal and spatial variations of nutrient loads corresponding to the variability of hydrological regimes and catchment characteristics.

## 2. Materials and Methods

### 2.1. HYPE Model

**Figure 1:**
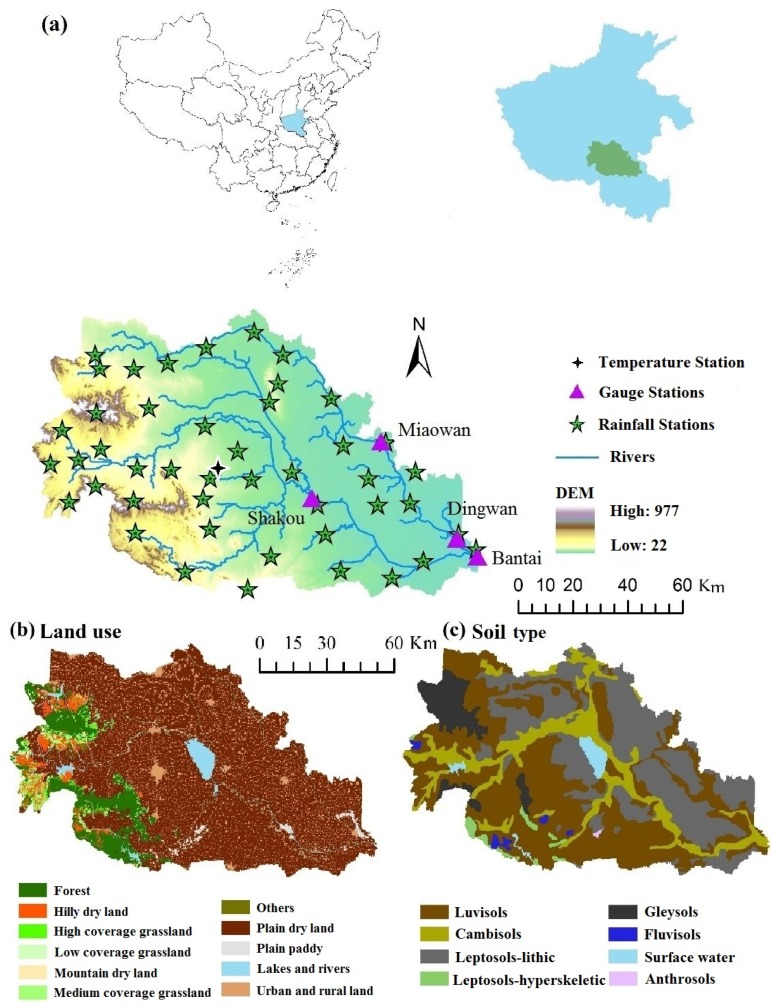
(**a**) Hong–Ru River Basin DEM and its location; (**b**) land use; and (**c**) soil type.

The HYPE model is developed by SMHI to simulate stream flow and water quality on their way from precipitation through soil, river, and lakes to river outlets over time [32]. The spatial division of the semi-distributed and dynamic model is linked to sub-basins and classes separated by land use and soil type, which is called land-use and soil type combination (SLC) in the paper. Each SLC is divided into a maximum of three soil layers. Each soil layer with a specific depth contributes to the runoff and water quality accumulations for a certain outlet (Figure 1). Most model parameters are related to land use or soil type; however, some parameters are global to a large region in a modeled area [32,34].

The HYPE model simulates snow accumulation and snowmelt, evapotranspiration, surface runoff, infiltration, macropore flow, percolation, interflow, tile drain flow, quick base flow, slow base flow, snow depth, frost depth, river delay, and damping in a hydrological process [10,32]. The accumulated water discharge is calculated because the model accommodates a river network and a river routing routine [36]. The main N and P sources and sinks in each SLC are illustrated to simulate these nutrients [34]. The sources include point and diffuse sources. Point sources from sewage treatment plants and industry are distinguished in this application. Diffuse sources are mainly contributed by agricultural land. Manure, local households, plant residues, and atmospheric deposition are also implicated. Inorganic nitrogen (IN), organic nitrogen (ON), particulate phosphorous (PP), and dissolved phosphorous (SP) concentrations in rivers and lakes can be simulated in the HYPE model at a daily time step. TN is calculated as the sum of IN and ON; TP is calculated as the sum of PP and SP. The mass balance calculations of mobile and immobile nutrient pools are performed for each element in each compartment [32,34]. SLCs are prepared by the user on the basis of a specific targeted area. SLCs are also related to crop data, including dates and amounts for fertilization and manure, timing of sowing and harvesting, and crop cover.

### 2.2. Study Area and Data

The Hong-Ru River Basin is a tributary of the Huai River Basin located in China’s Huang-Huai-Hai plain region. The Hong-Ru River Basin covers an area of 10,827 km^2^, which is a combination of Hong and Ru rivers, as they share the same outlet at the Bantai gauging station. A digital elevation model (DEM) was used to present the basin topography. The four discharge gauging stations (Miaowan, Dingwan, Shakou, and Bantai) with daily time step data from 2006 to 2010 were chosen in the Hong-Ru River Basin (Figure 1). Only three stations near Shakou, Dingwan, and Bantai were chosen to determine TN and TP concentrations from 2006–2010 because of insufficient recorded water quality. The recorded data include TN and TP concentrations of every other month from 2006 to 2008 but once a month from 2009 to 2010. Hong River originates at the Funiu Mountain in the southeastern part of Henan. The Ru River originates at the WuFeng Mountain and flows through three artificial reservoirs, namely, Banqiao, Suyahu, and Boshan. The three reservoirs have been considered in the model because these reservoirs have been described with detailed data, such as regulating volume, water depth below the threshold for olake (down to the mean depth), and other related normal variables. Elevation varies from 977 m to 22 m from headwater to the catchment outlet (Figure 1). Approximately 69% is covered by agricultural land, which yields two crops a year. Approximately 9% is covered by forest. Urban and rural lands located between agricultural lands accounted for 11% of the total area. Soil is dominated by luvisols (44%), and lithic covers 32%, mainly in the lowland areas (Figure 1). Cambisols are distributed almost the same way as the river networks.

Forty-five precipitation gauging stations were selected and relatively equally distributed. In this regard, the whole river basin can be reasonably monitored. The ungauged precipitations of some sub-basins were substituted by the nearby recorded rainfall. The sub-basin distributions are illustrated at Part 3.3.3 to enhance the understanding of the annual yields of TN and TP load simulations in each sub-basin. The mean annual precipitation in the whole catchment is 934 mm. The amount of precipitation in summer is higher than that in winter. The mean temperature is 15.7 °C, as indicated by the temperatures of all sub-basins from the same meteorological data in Zhumadian City Meteorological Bureau. Temperatures at >30 °C are detected in June, July, and August; the highest temperature is 34.7 °C. Conversely, temperatures at <0 °C are recorded in January and February; the lowest temperature is −6 °C. Agricultural land is treated as the main nutrient source contributing to eutrophication in stream water. The two main crops in lowland areas are winter wheat and summer maize. Some farmers prefer crop rotation over homogeneous crop management practices for peanuts, vegetables, and oil plants. According to a survey of interviews with 117 farmers by using a pre-constructed questionnaire regarding the basin, fertilizer inputs are the main eutrophication sources of the stream water. Approximately 300 kg N/ha and 120 kg P/ha are applied in October when winter wheat is planted, and 43 kg N/ha is added in February. Wheat is harvested by the end of May. Maize is planted subsequently. For maize, approximately 300 kg N/ha and 120 kg P/ha are applied in June, and 86 kg N/ha is added in July, which is about two months earlier before its harvest by the end of September. Table 1 defines the typical crop rotations for Hong Ru River Basin in China, crop data, simplified operation of each crop planting process, and the timing and amount of fertilizers. Crop rotations were mainly carried out on plain dry land. The mean TN and TP concentrations at the three water quality stations and the mean discharge at the four hydrological stations are listed in Table 2.

### 2.3. Model Setup and Parameter Calibration

The HYPE model was set up to simulate the discharge and stream water TN and TP concentrations in the Hong–Ru River Basin from 2005 to 2010. One year (2005) simulation was conducted for a preliminary model evaluation, which was ignored in the model evaluation. Model calibration (2006–2008) and validation (2009–2010) were performed as the input files were improved after one year of model warm up. The spatial and time series data prepared for the model setup are shown in Table 3. The calibration and validation of daily mean discharge and mean nutrient concentrations have been measured at the studied stations.

In this study, land use and soil were respectively reclassified into 11 and eight types on the basis of the original sources by using ArcGIS. The whole Hong-Ru River Basin was divided into 92 sub-basins, and 66 SLCs were defined on the basis of the descriptions of HYPE. The number of SLCs was greatly reduced by aggregating similar classes into larger groups that might exhibit similar properties. The driving data of daily precipitation and daily mean temperature of the discharge simulation in each sub-basin were prepared in accordance with the required format of HYPE; missing values were replaced with data from Zhumadian station, which represents the whole basin. The atmospheric depositions containing dry and wet depositions of N and P were filled with values from neighboring cities and provinces because of limited studies and monitoring data. The agricultural nutrient inputs were obtained from a field survey. Diffuse and point sources were prepared after the statistics from the Environmental Protection Agency of Zhumadian was analyzed.

Many parameters are related to hydrological and nutrient processes in the HYPE model. Some parameters are general, and other parameters are dependent on land use and soil type. Most of these parameters were derived from literature review and from previous modeling experiences, although only the most sensitive parameters were selected for calibration. Some parameters were held constant [10,30]. In this way, the risk of equifinality caused by the reciprocity between parameters can be reduced. The sensitive parameters obtained through manual sensitivity analysis with a one-factor-at-a-time approach [35,39] were then optimized using PEST, a nonlinear parameter estimator. PEST is a local search approach that uses the Gauss-Marquardt-Levenberg algorithm [40]. With PEST, a multi-site and multi-objective calibration approach was obtained; thus, hydrological and water quality parameters can be simultaneously calibrated using all available observed discharge and TN and TP concentrations from the gauging stations. Multi-site calibration was employed to explain the effects of the spatial variability of climate patterns, topography, land use, and soil type on hydrological and nutrient leaching processes and parameter sets [10]. Multi-objective calibration [10,41,42,43] is a more efficient and appropriate approach for parameter identification in hydrological and water quality modelling than other methods. In this study, relative composite sensitivity was obtained to determine the composite changes in model outputs caused by a fractional change in parameter values [44]. Objective functions (OF) were weighted to ensure that all the objective functions are of the same order of magnitude and are of similar significance in the search for the optimum. Global optimization criterion (GOC) was defined as the weighted sum of OFs. Each OF was calculated as the squared sum of weighted residuals. The definitions of GOC and OFs are expressed as Equations (1) and (2), respectively:

(1) $$OF=\underset{j=1,n}{\sum }{\left[x_{j,obs}-x_{j,sim}\right]}^{2}$$

and:

(2) $$GOC=\underset{i=i,m}{\sum }\omega_{i}OF_{i}$$

where *m* is the total number of the observation groups of the observed discharge and nutrient concentrations from the gauging stations, *n* is the total number of the measured discharge/nutrient concentrations at each gauging station, and ω is the weight of the related objective function.

Cubic spline interpolations were used in this study [22,24], and four main criteria [10,23,45,46,47], namely, coefficient of determination (R^2^), NSE, PBIAS, and root mean squared error (RMSE) observation standard deviation ratio (RSR), were used to evaluate the agreement between simulated (Sim) and observed (Obs) values of daily discharge and daily nutrient loads. The four criteria were determined on the basis of the following equations:

(3) $$PBIAS=\frac{\sum_{i=1}^{n}\left(Y_{i}^{sim}-Y_{i}^{obs}\right)*100}{\sum_{i}^{n}Y_{i}^{obs}}$$

(4) $$NSE=1-\frac{\sum_{i=1}^{n}{\left(Y_{i}^{sim}-Y_{i}^{obs}\right)}^{2}}{\sum_{i=1}^{n}{\left(Y_{i}^{obs}-\bar{Y_{i}^{obs}}\right)}^{2.}}$$

(5) $$RSR=\frac{RMSE}{STDEV_{obs}}=\frac{\sqrt{\sum_{i=1}^{n}{\left(Y_{i}^{sim}-Y_{i}^{obs}\right)}^{2}}}{\sqrt{\sum_{i=1}^{n}{\left(Y_{i}^{sim}-\bar{Y_{i}^{obs}}\right)}^{2}}}$$

(6) $$R^{2}=\frac{\sum_{i=1}^{n}\left(Y_{i}^{obs}-\bar{Y^{obs}}\right)\left(Y_{i}^{sim}-\bar{Y^{sim}}\right)}{\sqrt{\sum_{i=1}^{n}{\left(Y_{i}^{obs}-\bar{Y^{obs}}\right)}^{2}}\sqrt{\sum_{i=1}^{n}{\left(Y_{i}^{sim}-\bar{Y^{sim}}\right)}^{2}}}$$

According to the watershed simulation evaluation guidelines described in a previous study [28], model simulation at monthly intervals can be considered satisfactory if NSE > 0.5 and RSR < 0.7 and if PBIAS is ±25% for streamflow and PBIAS is ±70% for N and P. In this study, time step was set daily. Model simulations are typically poorer if simulations are evaluated at a shorter time step, that is, if the calculated values of the four criteria for daily model simulations are considered satisfactory according to the established value conditions, model performances need not be evaluated at monthly/yearly time step. Therefore, the preceding guidelines [7] were considered in the model evaluations at a daily time step as a priority.

## 3. Results and Discussion

### 3.1. Model Parameter Calibration and Validation

The parameter calibration results and the related physical interpretations are presented in Table 4. The most sensitive hydrological parameters are determined on the basis of the relative composite sensitivity of these parameters. For hydrological processes, the most sensitive parameters in a decreasing pattern are wcep (cambisols and gleysols-soil type dependent), rivvel (general parameter), and cevp (plain dry land–land use dependent). Wcep controls soil porosity, which influences the soil runoff in all of the soil layers. This finding indicates the important role of subsurface flow in forested mountainous areas [10] and in plain areas. Rivvel shows the maximum velocity during flooding, and this parameter affects the peak modeling of discharge. Cevp is sensitive because this parameter controls the potential evapotranspiration rate. For nutrient processes, sedimentation- and nutrient-production/decay-related sensitive parameters, such as sedimentation rate for ON in lakes (sedon), production/degradation in water for N (wprodn), sedimentation rate for PP in lakes (sedpp), and production/degradation in water for P (wprodp), affect TN and TP simulations. The relative composite sensitivities of sedpp and wprodp indicate that these parameters exhibit nearly the same effect on phosphorous-associated processes. This finding may be attributed to the difficulties in natural dephosphorization compared with denitrification.

The most sensitive parameter is denitrlu (plain dry land–land use dependent), which corresponds to denitrification in soil. This result is consistent with that described in previous studies [10], which revealed that the parameters related to N processes in soil are more sensitive than denitwrm, wprodn, and rivvel2 that are relevant to in-stream processes.

### 3.2. Hydrological Simulation

**Figure 2:**
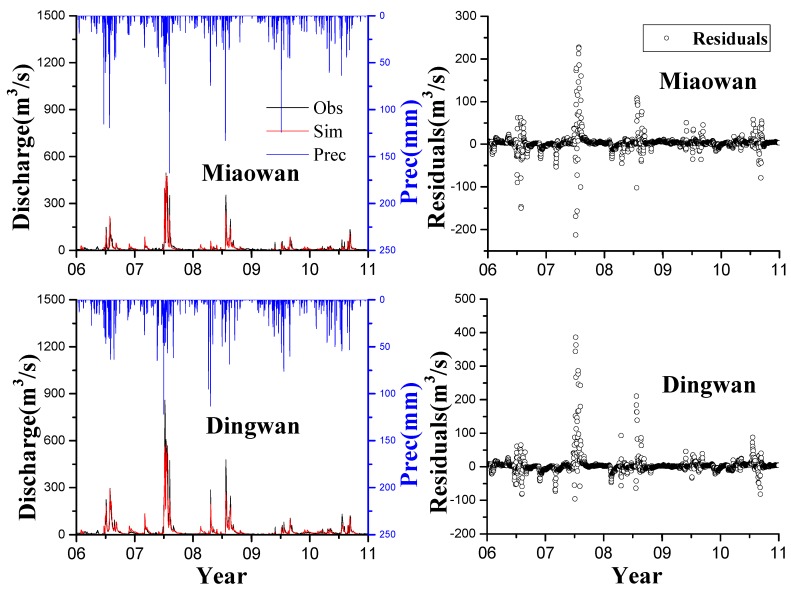
Daily discharge simulations at Miaowan and Dingwan stations in calibration (2006–2008) and validation (2009–2010) periods; residual plots are shown in the right panel.

**Figure 3:**
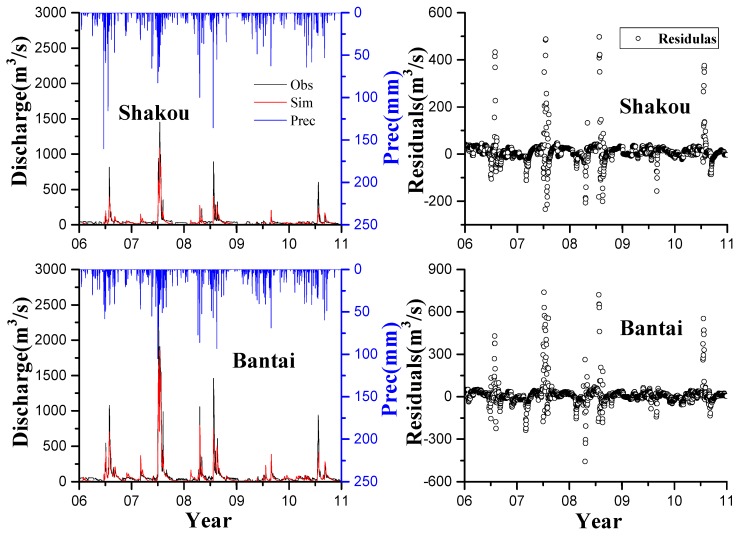
Daily discharge simulation at Shakou and Bantai stations in the calibration (2006–2008) and validation (2009–2010) periods; residual plots are shown in the right panel.

The discharge simulation results are separated into two sets. One set involves the comparison of two relatively low-discharge gauging stations, namely, Dingwan and Miaowan. The other set includes the comparison of two relatively high-discharge gauging stations, namely, Shakou and Bantai. Figure 2 and Figure 3 show that the model could capture the desired hydrological characteristics.

The highest observed discharge values at Dingwan and Miaowan stations were 858 and 496 mm, respectively. The highest observed discharge values at Shakou and Bantai stations were 1620 and 2310 mm, respectively. The HYPE model captured peak flow relatively well during both calibration and validation periods at the four stations, especially at Miaowan, Dingwan, and Bantai stations in July 2007. Some mismatches between simulated peak flow and observed peak flow is mainly due to the continuous model setup of a daily time step [48,49]. The residuals were also analyzed, and the positive values indicate that the observed discharge is higher than the simulated discharge; by contrast, the negative values show that the observed discharge is lower than the simulated discharge.

At the Mianwan and Dingwan stations, the residuals were intensely distributed around 0 as time passes. In summer in 2007, the residuals slightly fluctuated because of extreme climatological events, which changed groundwater base flow. This finding also occurred because the model did not well depict the complicated relationships between soil water and land use, especially in this case, which considered plain dry land [50,51].

In the validation period from 2009 to 2010 when precipitations were more than 40 mm, the peaks of the observed and simulated discharge were not presented well. This result is probably attributed to low soil water storage where agriculture lands are largely distributed [52]. With the low discharge trend, drought may occur in this area in the following years, as observed in subsequent years according to official reports from the local Environmental Protection Agency. However, most residuals were intensively distributed near 0, which indicates good discharge simulations. This finding can be similarly observed in other criteria, namely, NSE and PBIAS. The HYPE model well reproduced the temporal variations of discharge during both calibration and validation periods at all four stations. On the basis of the established guidelines, we can consider the model discharge simulation satisfactory. The calculated values of NSE from the four stations were >0.75 in both calibration and validation periods (Table 5). The lowest values were contributed by Shakou station with 0.74 and 0.79 of NSE in calibration and validation periods, respectively. These low values are obtained presumably because Shakou is the outlet of three upstream-regulated reservoirs, namely, Bantiao, Boshan, and Suyahu. These low values are also possibly attributed to the counterbalance of steep slope, forest land, and impermeable soil in the upstream mountain area [53]. The poorest simulation could be observed in RSR with 0.51 and 0.46 in each period; nevertheless, this finding was greater than the other values. For the PBIAS, most of the values were less than 15%; however, the PBIAS in the validation periods at Shakou station is 27.3%, which is three times higher than 9.1% in the calibration period. This result is probably contributed by the inter-annual climate variation related to precipitation and input data error [10]. The performances at Bantai station (NSE = 0.85, PBIAS = −4.2% in the calibration period and NSE = 0.94, PBIAS = 8.7% in the validation period) reveal that the simulated discharge is consistent with the measured discharge. Therefore, the model reproduced the temporal and spatial variations at low- and high-discharge stations in the agricultural land in calibration and validation periods. This finding should be further investigated through daily load simulations and monthly or yearly load change analysis.

### 3.3. TN and TP Simulations

#### 3.3.1. TN and TP Concentrations and Daily Load Simulations

**Figure 4:**
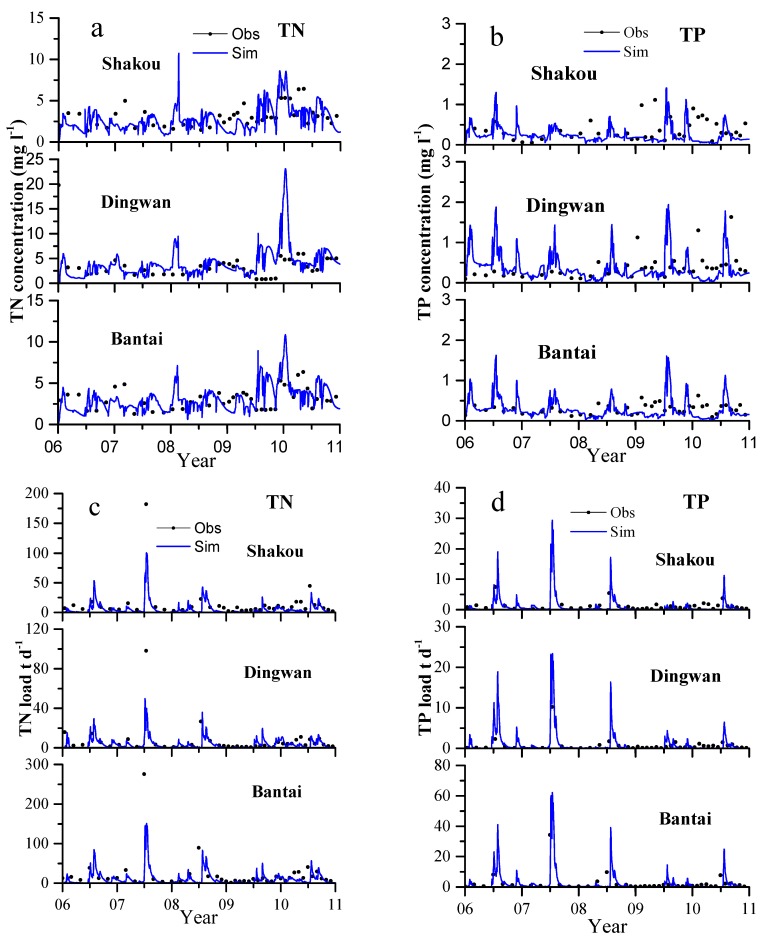
Observed and simulated daily TN and TP concentrations (**a**,**b**) together with the corresponding daily loads (**c**,**d**) during the calibration (2006–2008) and validation (2009–2010) periods.

The observed and simulated TN and TP concentrations and daily TN and TP load at the three gauging stations are illustrated in Figure 4a–d, respectively. HYPE represented the trend of the TN and TP concentrations well. The lowest performances were contributed by Shakou station (NSE = 0.52, PBIAS = −45.2% for TN concentrations and NSE = 0.57, PBIAS = −40.3% for TP concentrations) during calibration and validation periods. The model well reproduced the temporal variations during both calibration and validation periods at all three water quality stations. On the basis of the established guidelines, we can also consider the model nutrient load simulations satisfactory. The model results showed a similar pattern in terms of timing and magnitude at the three stations. This pattern is mainly influenced by similar agricultural landscape and hydrological factors, such as precipitation. The seasonal variations of the TN concentrations in the entire simulation period is insufficiently strong, and these variations are different from low and high concentrations in winter and summer. This difference is attributed to crop rotation included in the HYPE simulation [54,55,56]. In general, two crops are planted in a year in the studied river basin, whose condition is similar to those in many other areas in China. The overestimation and underestimation of TN and TP concentrations in the entire calibration and validation periods can be attributed to the uncertainty of bimonthly observed data because of insufficient financial support and personal constraints. In early 2010, the HYPE model failed to capture the high TN and TP concentrations. This phenomenon probably resulted from the low flow prediction [12]. The model revealed that the peaks of TP concentrations in each summer are greater than those of TN concentrations. This finding can be attributed to the simplified descriptions of the transport and transformation of P in the model.

As the outlet of Suyahu reservoir, Shakou station yielded weaker simulations of TN loads than Dingwan station. The performance of the model (Table 5) at Shakou is lower at NSE = 0.51 and 0.55 in calibration and validation periods, respectively, and at PBIAS = −48.6% and −20.6% than that at NSE and PBIAS calculated at Dingwan station. This lower performance influenced the model performance at Bantai station as the outlet of the whole river basin. Figure 4c shows that the model underestimated the TN load to a relatively large extent in summer in 2007. The underestimation occurred simultaneously with the extreme climatological events. The highest peak flow affected interflow and base flow, as well as residence time [57]. Denitrification in soil was over emphasized in this context [58]. Conversely, Figure 4 illustrates that the peaks of the modeled TP loads in each summer were overestimated compared with the observed values, which are the combined results of discharge and TP concentrations; these peaks probably contributed to the simplified transport and transformation of P in the soil. The model performance was affected entirely at the three water quality stations, especially at Dingwan and Bantai stations. The differences between the simulated daily TN and TP loads and the corresponding observations are mainly affected by the mismatches between observed and simulated discharge. This finding indicates that good hydrological simulation plays an important role in the reproduction of nutrient loads. Other studies have discovered similar findings [10,12]. The nutrient input from fertilizers among the studied area greatly contributes to the high evaluations of concentrations and loads in the river basin when the behaviors of the three representative water quality stations are summarized because agricultural landscape covers most areas of the basin. Thus, the environmental standards for TN and TP were a challenging target because of the effects of high nutrient loads.

#### 3.3.2. Monthly Yields of TN and TP Load Simulations

**Figure 5:**
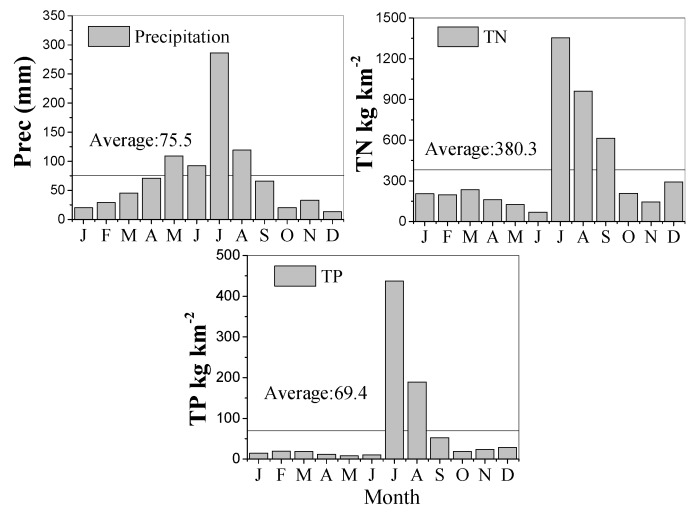
Average monthly total yields of TN and TP loads.

Figure 5 shows that the average values of the monthly TN loads exceeded the average level of 380.3 kg/km^2^ in July, August, and September; among these values, the highest load was simulated in July. This result corresponds well to the values in the months with a relatively high precipitation. The average total monthly precipitation values were 287 mm, 119 mm, and 66 mm in July, August, and September, respectively. The average total monthly loads in December, January, February, and March were relatively higher than those in April, May, and June. This difference can be contributed by the basic fertilizer inputs prepared for winter wheat in October in most areas of the agricultural land and additional fertilizer inputs at the end of February, although the corresponding precipitation was maintained at a relatively low level [59]. Another reason can be reduced denitrification and consumption by plants and microbes because of low water temperatures [60]. The lowest load was detected in June when winter heat was harvested. The contributions of fertilizers to catch crops, such as summer maize and peanuts, were mainly observed in June.

The trend of TP load yield fluctuations was similar to that of TN load yield fluctuations. The highest yields of 437.2 kg/km^2^, 189.4 kg/km^2^, and 52.4 kg/km^2^ were detected in July, August, and September, respectively. By contrast, the lowest yields of 8.2 kg/km^2^ and 10.5 kg/km^2^ were found in May and June, respectively. The yield in winter in December was 28.4 kg/km^2^, and this value was higher than those in February (19.2 kg/km^2^) and March (19.0 kg/km^2^). This difference is probably attributed to the reduced consumption of P and the decreased microbial population. In the first six months, the TP loads were lower than 19.2 kg/km^2^ in February, which could be the month when a low amount of P was used in winter wheat planting and less inputs were contributed by other nonpoint sources because point sources in the area were not the main contributors [61].

#### 3.3.3. Annual Yields of TN and TP Load Simulations in Each Sub-Basin

**Figure 6:**
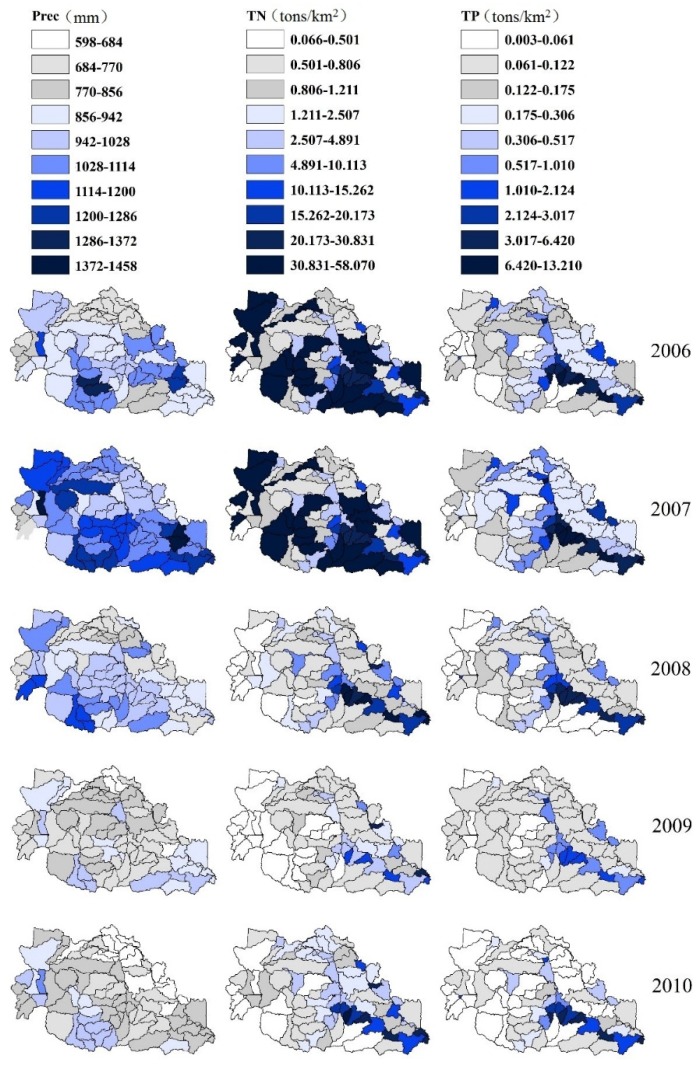
Spatial distribution of TN and TP load annual yields from 2006 to 2010.

**Figure 7:**
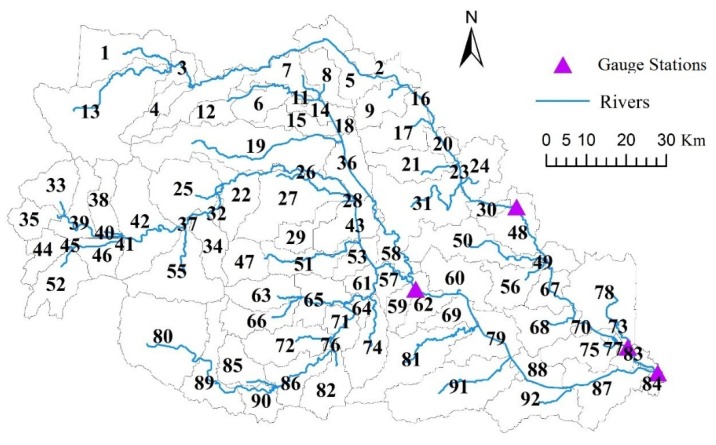
92 sub-basins and the main streams contained in the Hong–Ru River Basin based on ArcGIS.

The area-weighted TN and TP annual yields from 2006 to 2010 were determined (Figure 6) to investigate the spatial variations of nutrients within the Hong–Ru River Basin. The 92 contained sub-basins (Figure 7) represented the sub-basins with different soil types and land uses. The characteristics of annual yields in each sub-basin in this agricultural areas were obtained to further consider the agricultural management in a similar watershed in China. The total amount and fluctuation patterns of annual yields were investigated for each sub-basin.

The annual yields of the TN load varied from 0.066 tons/km^2^ to 58.07 tons/km^2^ for the five studied years. The initial TN load in 2006 ranged from 0.168 tons/km^2^ to 39.447 tons/km^2^. The sub-basins 33, 35, 44, 52, 55, and 88, which are located in the upstream mountain area, showed relatively low TN loads. This mountain area is dominated by forests and grasses (Figure 1). The other sub-basins, such as 91, 81, and 74, with low TN loads are located in the downstream of Shakou; the nutrients in this area are transported to the outlet of the river basin. The nutrients distributed along Hong River and Ru River were extremely high. The high nutrient load is contributed mainly by fertilizers, nonpoint sources from local household outflow, and point sources from water treatment plants [61,62,63]. The sub-basins near 59, where Shakou station is located, and the sub-basins near 84, where Bantai station is located, contained the highest average amount of TN in 2006. The annual area-weighted precipitation in 2006 was largely distributed in the upstream of Ru River and in the middle reaches of the Hong River; precipitation helps transport and transforms N; as a result, N sinks into the downstream outlets of the Ru and Hong rivers. In 2007, the TN loads sinking into the Ru River from the upland sub-basins 1, 13, 33, and 38 remarkably improved under extreme climatological circumstances in summer. This finding can be attributed to the high nutrient transport capacity through surface and subsurface corresponding to steep slope and highly permeable soils [10]. High TN loads could be detected in the plain area because agriculture is the dominant land use in these sub-basins. The transport and transformation of N were more active in the soil in terms of the available IN and ON loads [64,65,66]. The TN loads in 2008, 2009, and 2010 were likely normal and the annual precipitation was lower than 1028 mm in most sub-basins. High TN loads ranging from 10 tons/km^2^ to 58.07 tons/km^2^ were found on the downstream section of the Ru River. The manure from livestock and poultry is responsible for the high TN loads, as indicated by high fertilizer inputs.

The annual yields of TP loads varied from 0.003 tons/km^2^ to 13.210 tons/km^2^ during 2006–2010. The differences in the spatial distributions of TP loads for five years were relatively more normal than the distributions of TN loads. The highest amount of TP loads occurred along the Ru River. In 2006 and 2007, TP loads were relatively more than those in 2008, 2009, 2010. This finding indicates that high rainfall conditions were more significant; thus, this phenomenon indirectly describes temporal variation in the annual loads of TP through surface flow with sediments [7,67]. TP loads along Hong River were less than those along the Ru River. This difference was probably caused by low P sources from agricultural applications (low P applied during peanuts planting), manure input, and household release. Sub-basin 84 contained the highest amount of TP loads each year because TP leaked from the bottom, particularly the release of P during oxygen deficiency or the mixing of sedimented emissions in the model. PP was redistributed over time through sedimentation and resuspension.

### 3.4. Future Work

The HYPE model has been successfully used in agricultural lands with crop rotation to assess the temporal and spatial variations of water quality in the Hong Ru River Basin in China. However, the model should be further evaluated with appropriate tools to check its uncertainties because numerous data were obtained through literature review and many historical monitoring data were insufficiently described. The simplified descriptions of the model simulations, such as transport and transformation of P and processes during extreme climatological events of agricultural land, can also be further developed. Crop rotation with more crop plantings under different circumstances in one year is also a challenging work. The simplified structures of the HYPE model should be considered in practical applications because the model can be modified easily on the basis of specific characteristics.

## 4. Conclusions

HYPE was applied to the Hong–Ru River Basin with a daily time step. Discharge in this agricultural area from 2006 to 2010 was simulated; TN and TP concentrations and loads from 2006 to 2010 were also simulated. A multi-site and multi-objective calibration method was utilized with PEST to identify the relevant parameters. The most sensitive general parameters and land use/soil type parameters during hydrological simulations were wcep, rivvel, and cevp. The most sensitive general parameters and land use/soil type parameters during nutrient simulations were denitrlu, rivvel2, wprodn, wprodp, and sedpp. The optimized values were significantly satisfactory.

The HYPE model captured the temporal and spatial variations of discharge very well in both the calibration and validation periods at the four gauging stations containing high flow and low flow circumstances. Among the NSEs, the lowest was 0.74. However, the model underestimated the water balances at Shakou station and the catchment outlet Bantai by 27.3% and 8.7%, respectively. Shakou is very close to the outlet of the regulated reservoir Suyahu, and this station is also located downstream of the two other upstream reservoirs, namely, Bantiao and Boshan, which influence the flow through Shakou and Bantai stations to some extent. The underestimation was also observed because HYPE simplified the descriptions of the lakes within the catchment and disregarded the effects of reservoir management on the main rivers and land use; as a consequence, the model performance was reduced. The headwater contributes most of the total runoff in this studied catchment.

The HYPE model captured the temporal and spatial variations of TN and TP relatively well during both calibration and validation periods at the three gauging stations. The lowest NSE and PBIAS for TN are 0.51% and −48.6%, respectively. By contrast, the lowest NSE and PBIAS for TP are 0.54% and −38.6%. The seasonal variations of daily TN concentrations in the entire simulation period is not sufficiently significant, and these variations are different from the low and high concentrations in winter and summer reported in a previous study [10]. This result indicated that crop rotation changed the timing and amount of N output. The model generally overestimated TP concentrations and loads because of the simplified P processes. The model performance in the TN and TP concentrations in stream water was lower than that in their loads; this result showed that some nutrient processes, such as IN retention process and soluble reactive P absorbed by plants, should be improved in the model.

The average monthly TN and TP simulation yields revealed that nutrient outputs were mainly detected in summer by considering the corresponding discharge. The area-weighted TN and TP load annual yields showed that nutrient loads were extremely high along the Hong and Ru rivers, especially in the agricultural land. Annual area-weighted precipitation indirectly affected the transport and transformation of N to a greater extent than P.

Developed on the basis of Swedish characteristics, the HYPE model can be used in agricultural areas with crop rotation in China; this model can also be widely used worldwide. This model can be considered as a good decision-making tool of environmental protection agencies to predict discharge and enhance agricultural management with limited related data.

## Figures and Tables

**Table 1:** Crop rotations and fertilizer applications.

Crop Rotation	Crops	Date	Simplified Operation	Elemental Fertilizer (kg·ha^−1^)
Rotation 1	Winter wheat	7 October	Fertilization N:P	300:120
		8 October	Soil tillage	
		8 October	Planting	
		5 March	Fertilization N	43
		15 May	Harvest	
	Maize	15 June	Soil tillage	
		17 June	Fertilization N:P	300:120
		17 June	Planting	
		2 August	Fertilization N	84
		18 September	Harvest	
Rotation 2	Winter wheat	7 October	Fertilization N:P	300:120
		8 October	Soil tillage	
		8 October	Planting	
		5 March	Fertilization N	43
		15 May	Harvest	
	Peanuts	2 June	Soil tillage	
		4 June	Planting	
		4 June	Fertilization N:P	90:90
		15 September	Harvest	

**Table 2:** Mean TN and TP concentrations and discharge for the studied stations in 2006–2010.

Category	Miaowan	Dingwan	Shakou	Bantai
Mean TN concentrations (mg/L)	-	3.79	2.89	2.96
Mean TP concentrations (mg/L)	-	0.23	0.34	0.28
Mean discharge (m^3^/s)	15.87	23.24	42.48	74.34

**Table 3:** Spatial and time series input data for the HYPE model in the Hong-Ru river catchment.

Data Type	Data Description/Properties	Resolution	Source
Geographical data	Elevation	30 m	Chinese National Geomatics Center
	Stream network	-	
	Land use	25 m	
	Soil type	25 m	
Meteorological data	Daily precipitation	45 stations	Chinese Meteorological Administration
	Air temperature	1 station (Zhumadian)	Chinese Ministry of Water Resources
Agricultural practices	Manure and fertilizer, timing and amount for fertilization, sowing, and harvesting	-	Field survey (117 farmers)
Soil nitrogen content	Initial nitrogen storage	-	Literature review [37,38]
Sewage treatment plants	Water flow and TN and TP concentrations	-	Operating reports of sewage treatment plants

**Table 4:** Physical meanings, initial values and ranges, parameters’ sensitivity, and optimized values and confidence limits of key parameters.

Parameter	Physical Meaning	Initial Value	Initial Range	Relative Composite Sensitivity	Optimized Value	95% Confidence Limits
cevp						
Forest	Potential evapotranspiration rate ( mm·day^−1^·°C^−1^)	0.16	0.01–1	0.0014	0.17	0.141–0.195
Plain dry land		0.097	0.001–1	0.0075	0.0975	0.0967–0.0976
rrcs1						
Luvisols	Soil runoff coefficient for the uppermost soil layer (day^−1^)	0.4	0.01–1	0.0004	0.3	0.337–0.512
Leptosols-lithic		0.18	0.01–1	0.0005	0.15	0.135–0.19
wcep						
Luvisols	Effective porosity as a fraction	0.11	0.01–1	0.0009	0.113	0.108-0.124
Cambisols		0.0005	1 × 10^−5^–1	0.0087	0.000544	4.93 × 10^−4^–5.56 × 10^−4^
Gleysols		0.0002	1 × 10^−5^–1	0.010	0.00045	0.0001–5.2 × 10^−4^
rivvel	celerity of flood in watercourse (m·s^−1^)	1.202	0.1–10	0.0083	1.149	1.135–1.157
cevpcorr	Correction factor for evapotranspiration	0.1	0.01–1	0.0009	0.12	0.08–0.157
rivvel2	parameter for calculation of velocity of the water in the watercourse	0.94	0.01–1	0.0005	0.104	0.713–1.294
sedon	sedimentation rate of ON in lakes (m·d^−1^)	0.002	0.0001–1	0.0002	0.001	0.0029–0.0004
wprodn	production/decay of N in water (kg·m^−3^·d^−1^)	0.0001	1 × 10^−5^–1	0.0003	0.0003	8.2 × 10^−5^–0.0005
denitwrm	parameter for denitrification in main watercourse (kg·m^−2^·d^−1^)	0.005	1 × 10^−4^–1	0.00014	0.0059	0.0041–7.4 × 10^−4^
denitrlu						
plain dry land	parameter for denitrification in soil (d^−1^)	0.0228	1 × 10^−6^–1	0.0021	0.0246	0.0235–0.0293
sedpp	sedimentation rate of PP in lakes (m·d^−1^)	0.017	1 × 10^−4^–1	0.0008	0.013	0.011–0.028
wprodp	production/decay of P in water (kg·m^−3^·d^−1^; general)	0.01	0.001–1	0.0009	0.03	0.0036–0.040
pprelexp	parameter for PP from surface runoff and tile drains	1.8	0.1–10	0.0004	1.3	1.1–3.67

**Table 5:** Model evaluation statistics of daily discharge, daily TN, and TP load simulations at the studied gauging stations in calibration (2006–2008) and validation (2009–2010) periods (PBIAS has the unit percent, and the other criteria are unitless).

Variable	Calibration: 2006–2008				Validation: 2009–2010			
	NSE	R^2^	PBIAS	RSR	NSE	R^2^	PBIAS	RSR
Daily discharge								
Miaowan	0.87	0.90	3.2	0.39	0.86	0.92	1.8	0.42
Dingwan	0.85	0.94	3.3	0.38	0.83	0.93	8.3	0.41
Shakou	0.74	0.86	9.1	0.51	0.79	0.90	27.3	0.46
Bantai	0.85	0.95	−4.2	0.33	0.84	0.94	8.7	0.37
Daily TN load								
Dingwan	0.78	0.94	−11.5	0.54	0.85	0.92	−8.1	0.39
Shakou	0.51	0.81	−48.6	0.59	0.55	0.80	−20.6	0.65
Bantai	0.71	0.89	−33.4	0.55	0.75	0.87	−13.8	0.47
Daily TP load								
Dingwan	0.69	0.76	−28.5	0.52	0.79	0.81	−8.5	0.50
Shakou	0.54	0.62	−38.6	0.62	0.68	0.77	−12.4	0.59
Bantai	0.62	0.75	−29.8	0.54	0.74	0.80	−19.9	0.52
